# Comparative evaluation of diode laser fibrotomy versus intralesional dexamethasone plus hyaluronidase for moderate oral submucous fibrosis

**DOI:** 10.6026/973206300221239

**Published:** 2026-02-28

**Authors:** Vani Chappidi, Nagaraju Kamarthi, Farah Vaqar Ahmed Momin, Dheeraj Voulligonda, Naseer Mohammad

**Affiliations:** 1 Department of Oral Medicine and Radiology, Subharti Dental College, Swami Vivekanand Subharti University, Meerut, Uttar Pradesh, India; 2 Department of Periodontology, Sri Sai College of Dental Surgery, Vikarabad, Telangana, India; 3 Department of Oral Medicine and Radiology, Mamata Institute of Dental Sciences, Bachupally, Hyderabad, Telangana, India; 4 Department of Oral and Maxillofacial Surgery, Sri Sai College of Dental Surgery, Vikarabad, Telangana, India

**Keywords:** Oral submucous fibrosis (OSMF), diode laser, fibrotomy, intralesional steroids, hyaluronidase

## Abstract

Oral submucous fibrosis (OSMF) represents a chronic, progressive, potentially malignant disorder lacking consensus on optimal management strategies for moderate clinical stages. Therefore, it is of interest to compare diode laser fibrotomy (Group A) versus intralesional dexamethasone sodium phosphate plus hyaluronidase (Group B) in 30 patients with moderate (Group II-III) OSMF. Mouth opening and cheek flexibility measurements at baseline, 1, 3 and 6 months revealed statistically significant intragroup improvements in both groups (p < 0.001). Intergroup analysis demonstrated significantly greater functional gains with laser fibrotomy compared to intralesional therapy (p < 0.001), highlighting superior clinical efficacy. Thus, we show OSMF management by establishing diode laser fibrotomy as a minimally invasive, highly effective treatment option for moderate disease stages.

## Background:

Oral submucous fibrosis (OSMF) is a chronic, insidious, potentially malignant disorder predominantly affecting populations in South and Southeast Asia. The condition is characterised by juxta-epithelial inflammation followed by progressive fibrosis of the lamina propria and deeper connective tissues, resulting in reduced oral mucosal elasticity and gradual restriction of mouth opening [1, 2]. Areca nut chewing is recognised as the primary etiological factor, with contributory roles played by smokeless tobacco, nutritional deficiencies, genetic predisposition, autoimmunity and chronic inflammation [3, 4]. The clinical manifestations of OSMF include a burning sensation of the oral mucosa, intolerance to spicy food, progressive trismus, impaired speech and difficulty in mastication and swallowing. The malignant transformation rate of OSMF has been reported to range between 2% and 8%, emphasising the importance of early diagnosis and effective management [5, 6]. Management of OSMF is largely stage-dependent and aims to alleviate symptoms improve mouth opening and prevent malignant transformation. Conservative and medical management modalities such as habit cessation, antioxidants, corticosteroids, hyaluronidase, placental extracts and immunomodulators have been widely used, particularly in early and moderate stages. Intralesional corticosteroids combined with hyaluronidase are considered the most commonly employed medical therapy. However, prolonged treatment duration, inconsistent outcomes, patient non-compliance and potential systemic adverse effects limit their long-term efficacy [7, 8, 9-10]. Surgical intervention is usually reserved for advanced stages with severe trismus. Conventional surgical fibrotomy, though effective, is associated with increased intraoperative bleeding, postoperative pain, fibrosis and the need for reconstruction under general anaesthesia. The introduction of lasers in oral soft-tissue surgery has revolutionised the management of OSMF by offering a minimally invasive alternative with advantages such as reduced bleeding, precise tissue ablation, minimal postoperative fibrosis, faster healing and improved patient comfort [11, 12-13]. Therefore, it is of interest to compare the efficacy of diode laser fibrotomy and intralesional dexamethasone with hyaluronidase in the management of moderate oral submucous fibrosis.

## Materials and Methods:

## Study design and sample:

The present single-blinded randomized, controlled, clinical two arm parallel study was carried out in the Department of Oral Medicine and Radiology, Sri Sai College of Dental Surgery, Vikarabad, Telangana. 30 systemically healthy patients aged 18-65 years and diagnosed with moderate OSMF (Group II and III according to Khanna and Andrade classification) were recruited from the out-patient department of Oral Medicine and Radiology. The proposed study was approved by the Institutional Ethical Committee.

## Inclusion criteria:

[1] Clinically diagnosed Group II and III OSMF

[2] Minimum mouth opening ≥ 15 mm

## Exclusion criteria:

[1] Previous medical or surgical treatment for OSMF

[2] Clinical evidence of moderate/severe dysplasia or malignancy

[3] Presence of systemic illness or drug allergy

[4] Unwillingness to comply with follow-up visits

## Randomization:

Patients were randomly allocated into two groups using computer-generated randomisation:

[1] Group A: Diode laser fibrotomy (n = 15)

[2] Group B: Intralesional dexamethasone with hyaluronidase (n = 15)

## Treatment protocol:

A detailed case history, including habit history, was recorded for each patient. Written informed consent for participation in the study was taken from the patients before their recruitment into the study.

## Group A - laser fibrotomy:

Laser fibrotomy was performed under local anaesthesia, using a 980-nm solid-state diode laser designed for oral soft tissues (Model- Pioon 980 nm, 10 watts, Pioon Technology Co. Ltd, Wuhan, Hubei, China). The laser was employed as per the manufacturer's manual instructions. A metal handpiece with a disposable cutting fibre of 400 μm diameter was utilised in continuous wave emission mode at 2W power in contact mode. The total energy calculated was 4 J and the fluence was maintained at 50 J/cm2. An inverted Y-shaped incision in the buccal mucosa, with a depth of 2 mm, was made using an activated laser fibre tip. This will cut through all palpable fibrotic bands. In cases where fibrotic bands are present anteriorly, the incisions were extended anteriorly up to the commissures of the lips. Finger palpation was employed to break the fibrous bands and simultaneously, Heister's mouth opener was utilised to separate the cut fibrous bands, facilitating an increase in mouth opening. The average surgical time for the procedure was 20-30 minutes.

## Group B - intralesional steroid therapy:

Patients received biweekly intralesional injections of dexamethasone sodium phosphate (4 mg/mL) and hyaluronidase (1500 IU) for 4 weeks along with oral physiotherapy and topical steroids. The patients were administered submucosal intralesional injections of 1.5ml Dexamethasone sodium phosphate (Inj Decadron 4 mg/ml) and 1500 International Units of Hyaluronidase (Hynidase 1500 I.U.) mixed with 0.5 ml of 2% (1:80,000) Lignocaine Hydrochloride, once every 2 weeks (biweekly) for 4 weeks.

## Common therapy for Group A & Group B:

Patients were advised to perform oral physiotherapy, involving cheek ballooning and mouth-opening exercises utilising a bunch of ice-cream sticks, four times daily for 5 minutes each session, continuing for 6 months post-treatment. Systemic Antioxidant supplementation was given, specifically Cap Lycopene 4 mg, administered twice daily for 2 months. Furthermore, all patients were actively enrolled in the tobacco cessation clinic at the institute before the initiation of treatment.

## Clinical parameters evaluated:

[1] Mouth opening (MO): The measurement of mouth opening was made using vernier callipers, determining the distance between the mesio-incisal edge of the upper left central and the mesio-incisal edge of the lower left central incisor tooth in millimetres on maximum mouth opening. In patients with missing incisors, adjacent teeth were used as reference points. These measurements were made at baseline (MOb), at 1 month (MO1), at 3 months (MO3) and at 6 months (MO6) follow-up. Improvement in mouth opening (iMO) was calculated as the difference between successive follow-up visits and baseline.

[2] Cheek flexibility (CF): Cheek flexibility was assessed by measuring two points, each located one-third of the distance from the angle of the mouth on a line connecting the tragus of the ear and the angle of the mouth (v1). The subject was instructed to fully inflate their cheeks and the distance between the two points marked on the cheek was measured (v2). Cheek flexibility was calculated as the difference between v2 and v1. Cheek flexibility was measured at baseline (CFb), at 1 month (CF1), at 3 months (CF3) and at 6 months (CF6) follow-up, respectively. Improvement in cheek flexibility (iCF) was calculated as the difference between successive follow-up visits and baseline. All measurements were recorded by the same examiner to minimise inter-observer variability and were documented at baseline, 1 month, 3 months and 6 months post-treatment.

## Results:

All 30 patients completed the study with no loss to follow-up. The mean age of the patients in Group A was 34.4 and in Group B were 37.1. The male-to-female ratio in both groups was 13:2. Baseline comparison revealed no statistically significant differences between Group A (diode laser fibrotomy) and Group B (intralesional dexamethasone with hyaluronidase) for mouth opening and cheek flexibility, confirming baseline comparability. Both groups demonstrated statistically significant intragroup improvement in mouth opening at all follow-up intervals (p < 0.001) (Table 1). Group A showed an early and sustained increase, with mean mouth opening improving from 23.06 ± 4.63 mm at baseline to 32.63 ± 4.06 mm at 6 months follow-up (Table 2). Group B exhibited gradual improvement from 25.22 ± 5.62 mm at baseline to 30.53 ± 5.18 mm at 6 months follow-up. Intergroup comparison revealed significantly greater improvement in Group A at 6 months (p < 0.001) when compared to Group B (Table 3). Both groups showed statistically significant intragroup improvement in cheek flexibility over time (p < 0.001) (Table 2 & Table 3). Group A demonstrated greater improvement, increasing from 4.40 ± 1.55 mm at baseline to 7.40 ± 1.72 mm at 6 months (Table 2). Group B showed improvement from 4.67 ± 1.50 mm at baseline to 5.67 ± 1.72 mm at 6 months follow-up. Intergroup comparison showed significantly superior improvement in Group A (p < 0.001) (Table 3). Non-parametric analysis confirmed significant intragroup improvement for both clinical parameters in both groups. However, intergroup analysis demonstrated superior functional improvement in terms of mouth opening and cheek flexibility in the diode laser fibrotomy group (Table 4).

## Discussion:

Oral submucous fibrosis (OSMF) is a chronic, progressive, potentially malignant disorder characterised by juxta-epithelial inflammation, excessive collagen deposition and subsequent fibrosis of the oral mucosa. Progressive fibrosis leads to reduced tissue elasticity, resulting in trismus, burning sensation and compromised oral functions such as mastication, speech and swallowing [1, 2-3]. Management of moderate OSMF (Group II and III) remains a clinical challenge, as conservative therapy often provides limited benefit, while aggressive surgical intervention may not be justified. Baseline comparison between the two study groups revealed no statistically significant differences in mouth opening and cheek flexibility. This confirms that randomisation was effective and that both groups were clinically comparable before intervention. Establishing baseline equivalence is essential in interventional studies to ensure that observed differences at follow-up are attributable to treatment effects rather than pre-existing variability, as emphasised in earlier OSMF clinical trials by Pindborg *et al.* and Haider *et al.* [14-15]. Patients treated with diode laser fibrotomy demonstrated an early and sustained improvement in mouth opening, which can be attributed to the direct mechanical release of fibrotic bands combined with the biological advantages of laser surgery [16]. Diode lasers produce precise tissue incision with minimal collateral damage, reduced intraoperative bleeding and limited postoperative inflammation, thereby reducing secondary fibrosis during healing. These factors collectively contribute to improved long-term functional outcomes. Similar findings have been reported by Gupta *et al.*, Chaudhry *et al.*, Gondivkar *et al.*, Sharma *et al.* and Desai *et al.* who observed superior improvement in mouth opening following laser-assisted fibrotomy in moderate OSMF cases [12, 13, 17 and 18]. In contrast, intralesional steroid therapy produced a gradual improvement in mouth opening. Corticosteroids suppress fibroblast proliferation and collagen synthesis, while hyaluronidase enzymatically degrades hyaluronic acid, increasing tissue permeability. Although this combination reduces fibrosis biochemically, it does not provide immediate mechanical release of established fibrotic bands, which may explain the comparatively lesser improvement in mouth opening observed in this group. These findings are consistent with previous study by James *et al.* [10]. The superior improvement in cheek flexibility following laser fibrotomy may be attributed to complete transection of fibrotic bands and reduced postoperative scar formation. Laser-induced photothermal effects are known to promote favourable wound healing with minimal contraction, thereby enhancing mucosal elasticity. Similar observations have been reported by recent studies conducted by Gupta *et al.* and Desai *et al.* who have emphasised improved mucosal pliability following surgical and laser-based interventions [17, 18]. Intralesional therapy, while effective in reducing inflammation and early fibrosis, may have a limited impact on long-standing dense fibrotic bands, thereby resulting in comparatively modest improvement in cheek flexibility [19]. The findings of the present study suggest that while both treatment modalities are effective in the management of moderate OSMF, diode laser fibrotomy offers superior functional improvement, in terms of mouth opening and cheek flexibility, compared to intralesional steroid therapy. These results support a stage-based or individualised treatment approach, wherein laser fibrotomy may be preferred for patients with moderate fibrosis requiring functional improvement [20, 21, 22-23]. The strengths of the present study include a randomised design, standardised measurement of clinical parameters, use of validated assessment tools and complete follow-up of all participants. However, the study is limited by a relatively small sample size and a follow-up duration of six months. Long-term follow-up studies with larger sample sizes are required to assess disease recurrence and sustained functional outcomes.

## Conclusion:

We show that both diode laser fibrotomy and intralesional dexamethasone with hyaluronidase are effective treatment modalities for moderate oral submucous fibrosis. However, diode laser fibrotomy provides superior functional improvement in terms of mouth opening and cheek flexibility. Hence, diode laser fibrotomy may therefore be considered a preferred minimally invasive therapeutic option in the management of moderate OSMF.

## Figures and Tables

**Table 1:** Analysis of demographic data and clinical parameters among group A and group B patients at baseline

**Variable**	**Group A**	**Group B**	**P-value**
Age	34.40 ± 7.95	37.07 ± 11.9	0.65 (NS)
Gender (Male:Female)	13:02	13:02	1.00 (NS)
Mouth Opening (Interincisal distances in mm)	23.06mm ± 4.63	25.22 ± 5.62	0.06 (NS)
Cheek Flexibility (in mm)	4.40 ± 1.55	4.67 ± 1.50	0.18 (NS)
S= significant (p < 0.05);
NS= not significant (p > 0.05)

**Table 2:** Intra-group comparison of clinical parameters in group A using the friedman test

**Clinical Parameter**	**Variable**	**N**	**Mean**	**SD**	**χ^2^ value**	**df**	**p value**	**Post-hoc analysis**
Mouth Opening (IID in mm)	MO b	15	23.06	4.63	38.72	3	<0.001 (S)	MO b > MO 1>MO3 > MO6
	MO 1	15	28.86	3.64				
	MO 3	15	30.44	3.98				
	MO 6	15	32.63	4.06				
Cheek Flexibility (CF IN mm)	CF b	15	4.4	1.55	31.64	3	<0.001 (S)	CF b > CF 1 > CF 3 > CF 6
	CF 1	15	6.27	1.67				
	CF 3	15	6.87	1.96				
	CF 6	15	7.4	1.72				
S = Significant (p < 0.05);
NS = Not significant (p > 0.05).
Statistical analysis performed
using the Friedman test with the
Wilcoxon signed-rank post-hoc analysis

**Table 3:** Intra-group comparison of clinical parameters in group B using the friedman test

**Clinical Parameter**	**Variable**	**N**	**Mean**	**SD**	**χ^2^ value**	**df**	**p value**	**Post-hoc analysis**
Mouth Opening (IID in mm)	IID b	15	25.22	5.62	29.85	3	<0.001 (S)	MO b > MO 1>MO3 > MO6
	IID 1	15	26.81	5.52				
	IID 3	15	28.97	5.32				
	IID 6	15	30.53	5.18				
Cheek Flexibility (CF in mm)	CF b	15	4.67	1.5	14.92	3	0.002 (S)	CF b > CF 1 > CF 3 > CF 6
	CF 1	15	5	1.56				
	CF 3	15	5.47	1.6				
	CF 6	15	5.67	1.72				
S = Significant (p < 0.05);
NS = Not significant (p > 0.05).
Statistical analysis performed
using the Friedman test with the
Wilcoxon signed-rank post-hoc analysis

**Table 4:** Comparison of change in clinical parameters between group A and group B using mann-whitney U test

**Variable**	**N (Group A)**	**Mean (Group A)**	**SD**	**N (Group B)**	**Mean (Group B)**	**SD**	**Mann-Whitney U**	**P value**
C-MOb-MO1	15	5.8	1.21	15	1.59	1.08	52	0.001 (S)
C-MOb-MO3	15	7.38	1.34	15	3.75	1.29	48.5	<0.001 (S)
C-MOb-MO6	15	9.57	1.46	15	5.31	1.41	45	<0.001 (S)
C-CFb-CF1	15	1.87	0.52	15	0.33	0.41	50	0.002 (S)
C-CFb-CF3	15	2.47	0.61	15	0.8	0.55	46	<0.001 (S)
C-CFb-CF6	15	3	0.66	15	1	0.6	42	<0.001 (S)
S = Significant (p < 0.05);
NS = Not significant (p > 0.05)
